# Postsynaptic Calcium Extrusion at the Mouse Neuromuscular Junction Alkalinizes the Synaptic Cleft

**DOI:** 10.1523/JNEUROSCI.0815-23.2023

**Published:** 2023-08-09

**Authors:** Ryan J. Durbin, Dante J. Heredia, Thomas W. Gould, Robert B. Renden

**Affiliations:** ^1^Integrative Neuroscience Graduate Program, University of Nevada, Reno, Reno, Nevada 89557; ^2^Department of Physiology and Cell Biology, University of Nevada, Reno, School of Medicine, Reno, Nevada 89557

**Keywords:** acetylcholine, mouse, neuromuscular junction, pH, synapse, synaptic transmission

## Abstract

Neurotransmission is shaped by extracellular pH. Alkalization enhances pH-sensitive transmitter release and receptor activation, whereas acidification inhibits these processes and can activate acid-sensitive conductances in the synaptic cleft. Previous work has shown that the synaptic cleft can either acidify because of synaptic vesicular release and/or alkalize because of Ca^2+^ extrusion by the plasma membrane ATPase (PMCA). The direction of change differs across synapse types. At the mammalian neuromuscular junction (NMJ), the direction and magnitude of pH transients in the synaptic cleft during transmission remain ambiguous. We set out to elucidate the extracellular pH transients that occur at this cholinergic synapse under near-physiological conditions and identify their sources. We monitored pH-dependent changes in the synaptic cleft of the mouse levator auris longus using viral expression of the pseudoratiometric probe pHusion-Ex in the muscle. Using mice from both sexes, a significant and prolonged alkalization occurred when stimulating the connected nerve for 5 s at 50 Hz, which was dependent on postsynaptic intracellular Ca^2+^ release. Sustained stimulation for a longer duration (20 s at 50 Hz) caused additional prolonged net acidification at the cleft. To investigate the mechanism underlying cleft alkalization, we used muscle-expressed GCaMP3 to monitor the contribution of postsynaptic Ca^2+^. Activity-induced liberation of intracellular Ca^2+^ in muscle positively correlated with alkalization of the synaptic cleft, whereas inhibiting PMCA significantly decreased the extent of cleft alkalization. Thus, cholinergic synapses of the mouse NMJ typically alkalize because of cytosolic Ca^2+^ liberated in muscle during activity, unless under highly strenuous conditions where acidification predominates.

**SIGNIFICANCE STATEMENT** Changes in synaptic cleft pH alter neurotransmission, acting on receptors and channels on both sides of the synapse. Synaptic acidification has been associated with a myriad of diseases in the central and peripheral nervous system. Here, we report that in near-physiological recording conditions the cholinergic neuromuscular junction shows use-dependent bidirectional changes in synaptic cleft pH—immediate alkalinization and a long-lasting acidification under prolonged stimulation. These results provide further insight into physiologically relevant changes at cholinergic synapses that have not been defined previously. Understanding and identifying synaptic pH transients during and after neuronal activity provides insight into short-term synaptic plasticity synapses and may identify therapeutic targets for diseases.

## Introduction

Dysregulation of brain pH has been implicated in neurodegenerative diseases and psychiatric disorders (Dodge et al., 2013; Hagihara et al., 2018). Low pH in the synaptic cleft inhibits L-type voltage-gated Ca^2+^ channels (VGCC) and NMDA receptors (NMDARs), and activates acid-sensing ion channels, shaping neurotransmission (Chesler, 2003; Makani and Chesler, 2007; Cho and Von Gersdorff, 2014; González-Inchauspe et al., 2017; Alijevic et al., 2020). Characterizing proton transients and extracellular buffering during neurotransmission and identifying their downstream effects will yield insight into synaptic function and provide targets for treatment of neurologic disease; however, mechanisms modulating synaptic pH changes are only partially known and have only been studied at a few synapses.

Experimentally, the orientation of pH transients differs by synapse. Ribbon synapses, specialized for release of large quantities of proton-packed glutamatergic synaptic vesicles, acidifies the synaptic cleft during activity, decreasing subsequent release of vesicles by inhibiting Ca^2+^ channels (DeVries, 2001; Palmer et al., 2003; Nouvian et al., 2006; Cho and von Gersdorff, 2014). However, at conventional glutamatergic synapses on CA1 hippocampal pyramidal neurons activity causes bulk extracellular alkalization, attributed to Ca^2+^ extrusion by the plasma membrane ATPase (PMCA; Makani and Chesler, 2010). Similarly, other glutamatergic synapses like the calyx of Held and the *Drosophila* larval neuromuscular junction (NMJ) show activity-dependent alkalization of the synaptic cleft, correlated with Ca^2+^ influx (Stawarski et al., 2020). The PMCA imports 2 H^+^ for every Ca^2+^ extruded (Thomas, 2009) and is active on both sides of the synapse following neurotransmission activity when intracellular Ca^2+^ levels are elevated. Thus, the PMCA acts to link Ca^2+^ extrusion at synaptic compartments leading to increased synaptic cleft pH (Lnenicka et al., 2006; Feghhi et al., 2021).

Evidence for acidification or alkalization at synapses using other transmitter types is limited. At the cholinergic mammalian neuromuscular junction, a biphasic intracellular pH transient was identified in presynaptic motor neuron terminals innervating the mouse fast-twitch levator auris longus (LAL) muscle in response to prolonged stimulation (Zhang et al., 2010). A brief acidification transient linked to intracellular Ca^2+^ concentration was followed by alkalization because of surface activity of the vacuolar-type H+-ATPase (vATPase) at the plasma membrane, pumping protons from the cytosol into the cleft (Zhang et al., 2010). Based on these findings, if an extracellular pH transient is assumed to mirror intracellular changes, a brief cleft alkalization followed by long acidification would be expected. This premise is supported by interstitial acidification that occurs during muscle fatigue (Juel, 2008). However, Blaustein et al. (2020) found synaptic extracellular pH at the LAL NMJ acidified due brief high-frequency stimulation, attributing the acidification to the vesicular release of protons. Because alkaline pH transients are predicted, but acid transients were measured, ambiguity regarding pH transients in the NMJ synaptic cleft remains. Most, if not all, previous studies at the NMJ typically block nicotinic acetylcholine receptors (nAChRs) to inhibit contraction and movement artifact. As a result, this manipulation blocks Ca^2+^ influx through nAChRs and the large amount of Ca^2+^ released from intracellular stores in the muscle during synaptic activation (Endo, 2009; Calderón et al., 2014). Given the established link between Ca^2+^ and pH, it is likely that synaptic cleft pH transients measured this way are muted because a postsynaptic contribution to the synaptic cleft pH transient is blocked. Notably, at the *Drosophila* NMJ, an activity-dependent increase in synaptic cleft pH was eliminated when postsynaptic receptors were blocked (Stawarski et al., 2020).

Here, we examined pH changes in the synaptic cleft of the LAL using pHusion-Ex, a pesudoratiometric genetically encoded fluorescent probe expressed in muscle and positioned in the extracellular leaflet of the NMJ endplate facing the extracellular space (Stawarski et al., 2020). To identify the contribution of muscle activity to the synaptic pH transient, we blocked muscle contraction with the muscle-selective myosin inhibitor 3-(N-butylethanimidoyl)−4-hydroxy-2H-chromen-2-one (BHC), leaving the postsynaptic transduction cascade intact (Heredia et al., 2016). We found large, activity-dependent pH shifts tied to postsynaptic buffering of Ca^2+^ released from intracellular stores, which result in transient alkalization, followed by prolonged acidification following strong stimulation.

## Materials and Methods

### Animals

All animals in these experiments were used in compliance with National Institutes of Health guidelines and approved animal protocols at University of Nevada, Reno. WT C57BL/6 mice were acquired from Charles River Laboratories (C57BL/6NCrl, strain #027). Mice of both sexes were used between 2 and 8 months of age. CAGGS-GCaMP3 transgenic mice show robust expression in skeletal muscle, as reported in Heredia et al. (2016).

### Injection of pHusion-Ex and preparation of the LAL

One-month-old mice were anesthetized with isoflurane (∼3.5% isoflurane with 2 L per min O^2^ flow rate) and maintained at a surgical plane for the duration of the surgery. Hair was removed with clippers and depilatory cream between the ears from the scalp to the most rostral edge of the scapulae, and the scalp was disinfected with 70% ethanol. The animal was placed on a heating mat (37°C) in a stereotaxic surgery platform and fixed in place via a bite bar and ear bars. A 1–1.5 cm incision in the skin was made on the dorsal surface of the scalp ∼0.5 cm to the right of the midline starting from the base of the skull. Forceps were used to gently peel back the skin on the right side of the incision, exposing the LAL. Fifteen to 20 μl of adeno-associated virus (AAV)9-pHusion-Ex (Penn Vector Core) was injected inferior to the right LAL, expelling virus as superficially as possible. Animals were then sutured and allowed to recover on a heating pad. In some experiments, similar volumes of virus were injected subcutaneously through the skin in the region of the LAL. We found better infectivity and greater expression of the probe in the LAL by this second approach.

After at least 2 months postinjection, animals were killed via inhaled isoflurane. Heart removal was used as secondary euthanasia. The right LAL was dissected as described previously (Burke et al., 2018), with motor nerve intact (∼3–5 mm of the nerve lifted from the connective tissue) and pinned in a SYLGARD dish in physiological buffer containing the following (in mm): 127 NaCl, 5 KCl, 2 CaCl_2_, 1 MgSO_4_, 10 NaHCO_3_, 1 Na_2_HPO_4_, and 11 glucose, 300 mOsm ± 10 mOsm, adjusted using sucrose. Solutions were bubbled with 95% O_2_/5% CO_2_, pH 7.3 ± 0.1, adjusted with NaOH after gassing, unless indicated otherwise. The motor nerve was stimulated with a parallel bipolar electrode, and contraction of the LAL was observed to ensure the NMJ preparation was intact and responsive to nerve stimulation. Successful dissections were then transferred to an upright microscope for imaging.

### Wide-field fluorescence imaging

Wide-field images were taken of the pinned preparation on an Axio Examiner A1 upright microscope (Zeiss) with a 40× water immersion objective (0.8 NA). Nerve stimulation was delivered by a Grass Technologies S48 Stimulator and a Grass Instruments SIU5A Stimulus Isolation Unit (4–8 V, 0.3 ms duration, biphasic pulse) to a suction electrode containing the cut nerve end. The preparation was continuously superfused with physiological saline at ∼4–8 ml/min, constantly bubbled with 95% O_2_/5% CO_2_, and temperature was maintained at 32°C using an inline heater (TC-324C, Warner Instruments). A peristaltic pump was used to recycle the bath solution to a 60 ml reservoir for the duration of the recordings. All solutions were maintained at pH 7.3 after gassing, unless indicated otherwise.

Fluorescence illumination of pHusion-Ex, a pseudoratiometric probe with tandem seGFP and pH-insensitive FusionRed tethered to the extracellular face of muscle (Stawarski et al., 2020), was controlled by an LED light engine (Lumencor SOLA) attenuated 80% by a neutral density filter. Preparations were illuminated with a dual-band filter set (FITC/TRITC, catalog #59004, Chroma). Red and green emission channels were imaged simultaneously using a W-VIEW GEMINI (Hamamatsu) equipped with a Beamsplitter FT 560 (Zeiss) separating the red and green channels. The red channel was additionally filtered (605/30 nm) to decrease potential bleedthrough between channels. Images were acquired by a Prime 95B sCMOS camera (Teledyne Photometrics) controlled by VisiView Software (Visitron Systems). Illumination, stimulation, and camera triggers were provided by transistor–transistor logic pulses from an Axon 1440 Data Acquisition System board controlled by pCLAMP 10.0 software. NMJs were identified using brightfield illumination, then confirmed by expression of pHusion-Ex at the endplate. Successful stimulation with the suction electrode was verified by muscle twitch before drug incubation. Imaging sequences included a fast acquisition rate (10–20 Hz) during stimulation followed by slower acquisition (0.1 Hz) during recovery, ending at ∼600 s. Stimulus trains were delivered at 50 Hz for either 250 ms, 5 s, or 20 s; 250 ms trains were to represent small and fast activity in the muscle, 5 s trains were to represent more strenuous activity in the muscle, and 20 s trains were to represent fatiguing stimuli. We found 20 s was a sufficiently fatiguing stimulus as failures were observed when imaging GCaMP3 transients in the LAL (data not shown). Images were acquired with interleaved no-stimulus controls. Images were captured either with 100 ms exposure (for the 20 s and 5 s stimuli) or 50 ms exposure (250 ms stimuli). Twenty minutes of rest was given between acquisition sequences. Only one stimulation condition (i.e., 20 s trains, 5 s trains, or 250 ms trains) was introduced in a preparation. An additional assay was also conducted to partially replicate the work in Blaustein et al. (2020) under physiological conditions. In short, preparations were incubated with the nAChR inhibitor tubocurarine and stimulated for 250 ms at 50 Hz. Image stacks were acquired continuously with 20 ms exposure and were looped 20 times with a 30 s rest between trains. Images were acquired with interleaved no-stimulus controls.

LALs expressing GCaMP3 were imaged with 50 ms exposure for 40 s, and stimulation conditions were gathered from the cell either in the order of no stimulation, 20 s, 5 s, 250 ms, or reverse order. A rest of ∼20 min was given in between stimulations.

### Image analysis

Dual-color fluorescent images (12- or 16-bit depth) were processed via ImageJ software. A square region of interest (ROI) was created to fit the NMJ endplate in each channel, and the background was subtracted using an ROI of the same size. Green channel/red channel ratios were created and normalized to the ratio of prestimulus time points. Stimulus trains, and no stimulus controls, were averaged within each condition and then subtracted to avoid photobleaching artifacts. In the case of the tubocurarine experiments, the no stimulus control values were subtracted from their corresponding values in the subsequent stimulus train. Intensity ratio minima and maxima were calculated between the onset of stimulation (0 s) to 1 min after stimulation.

GCaMP images were processed similarly, with average fluorescence within a square ROI surrounding the NMJ background subtracted and normalized to the average of the fluorescence values in both the first 5 s before the stimulus and 5 s at end of acquisition when signal was at baseline.

### pH Calibration

LALs were incubated with three different pHs of highly buffered physiological saline (pH 7.2, 6.7, and 7.7 in 20 mm HEPES and 10 mm bicarbonate) in a static bath for 30 min, following an approach used previously (Stawarski et al., 2020). Ten images were taken of an NMJ at 100 ms exposure. The preparation was then perfused with a new pH buffered solution for 20 min. After 10 min of rest in static bath, the NMJ was imaged again, and the process was repeated until all conditions were gathered. The buffer incubation order was either pH 7.2–6.7–7.2–7.7–7.2, or pH 7.2–7.7–7.2–6.7–7.2. Values in each condition were aggregated, and the green/red fluorescence ratios were taken. The ratios were then normalized to pH 7.2, and a linear regression was used to relate change in pH to change in normalized fluorescence ratios. This linear regression was used in the subsequent experiments to convert changes in the green/red ratio to pH measurements. Calibration data from previous reports confirmed we were measuring within the linear range of SE-pHluorin (Sankaranarayanan et al., 2000; Stawarski et al., 2020).

### Chemicals

In separate experiments, BHC or µConotoxin GIIB (µCono; catalog #C-270, Alomone Labs) was used to inhibit muscle contraction (Hill et al., 1996; Li and Tomaselli, 2004). BHC was synthesized and purified as reported previously (Heredia et al., 2016; Brawley et al., 2020), kept at 200 mm stock solution at −20°C, diluted 2:1 in DMSO before addition to physiological buffer, and used at 400 μm final concentration. A pinned LAL preparation was incubated for 30 min in a 4 ml static bath of physiological solution with 400 μm BHC or 2.5 μm µCono. Before imaging, the preparation was perfused with 60 ml of recycled physiological solution continuously bubbled with 95% O_2_/5% CO_2_ and heated to 32°C ± 2°C for 20 min. The BHC and µCono in the static bath were recycled to maintain inhibition at 2.7 um BHC or 16.7 nm uCono, respectively. While maintaining the perfusion and temperature of the bath, the LAL was stimulated at 50 Hz for various durations and imaged over the course of 600 s. Tubocurarine was purchased from MilliporeSigma (catalog #T2379; Blaustein et al., 2020; Zhang et al., 2010), acetazolamide was purchased from MilliporeSigma (catalog #A6011; Bertone et al., 2017), and caffeine was purchased from MilliporeSigma (catalog #C0750; Neyroud et al., 2019). Caloxin 1b1 was purchased from AnaSpec (catalog #AS-64236; Pande et al., 2006, 2011).

### Immunohistochemistry

Adapted from Wright et al. (2011), the dissected LAL was fixed in 4% paraformaldehyde, blocked with 0.1 m glycine in 1× PBS, permeabilized with methanol, and then blocked and permeabilized a second time with 1% fish gelatin, 0.5% Triton X-100, 0.025% NaN_3_, in PBS. After washing out blocking buffer, anti-pan-PMCA (1:1000; catalog #MABN1802, MilliporeSigma) and anti-vesicular acetylcholine transporter (vAChT) primary antibodies (1:1000; catalog #618250, MilliporeSigma), were incubated at 4°C overnight in PBS. After several washes with PBS, muscle was incubated overnight with Alexa Fluor 647 (1:500; catalog #105-605-003, Jackson ImunoResearch), Alexa Fluor 594–conjugated α-bungarotoxin conjugate (1:200; catalog #00007, Biotium), and STAR Green secondary antibodies (1:500; STGREEN-1001, Abberior) at 4°C. After being washed with PBS, tissues were mounted under coverslips in Prolong Glass Antifade (catalog #P36980, Thermo Fisher Scientific) and cured for >5 d. Super-resolution images were taken on a Leica STELLARIS 8 laser scanning confocal microscope. Three-color tau-stimulated emission depletion (STED) images were taken as sequential image stacks from longer to shorter wavelengths to avoid bleaching. Depletion lasers were set at 775 nm = 100% (Alexa Fluor 647), 775 nm = 50% (Alexa Fluor 594), and 592 nm = 37% (STAR Green). Three-dimensional tau-STED power was set to 60%. At these settings, lateral resolution was 60–100 nm, verified with 20 nm carboxylate-modified fluorescent beads (FluoSpheres, Thermo Fisher Scientific; data not shown).

For imaging surface expression of GFP, an LAL infected with pHusion-Ex was fixed and processed as above, but without permeabilization via methanol or Triton X-100. Tissue was incubated in anti-GFP primary antibody (1:1000; catalog #600-101-215, Rockland) at 4°C overnight, washed, and then incubated with Alexa Fluor 647 secondary antibody at 4°C overnight. After washing, tissue was mounted in SlowFade Diamond (catalog #S36967, Thermo Fisher Scientific), and images taken on an Olympus FV1000 laser scanning confocal microscope. Confocal and STED images were prepared and presented with Fiji/ImageJ software.

### Quantitative RT-PCR

Both LALs from an individual animal were rapidly dissected and dropped into 1 ml of TRIzol, and a bead homogenizer was used to lyse the tissue. Phenol/chloroform phase separation was used to extract RNA from the samples. A cDNA library was built using a qScript cDNA Synthesis Kit (catalog #95047, QuantaBio). The cDNA was then diluted to ∼500 ng/μl. Performing a standard curve qPCR, each sample was serially diluted three times and plated in triplicate for all PMCA isoforms (PMCA1, PMCA2, PMCA3, PMCA4) and GAPDH. Gene-specific primers (Table 1) were added to their respective wells with 2 μl of sample dilutions and Fast SYBR Green Master Mix (catalog #4385612, Thermo Fisher Scientific) in 96-well plates. Negative controls with no template cDNA were also included in triplicate for every primer included on each plate. The qPCR was run on QuantStudio 3 and 5 Real-Time PCR System Software (Applied Biosystems). Standard curves relating 1og_10_ transformed dilutions and cycle count were generated using linear regression. Data with cycle count values >35 were excluded.

**Table 1.** qPCR primers

Gene name	Sequence	GenBank accession number
Gapdh	F-GCCGATGCCCCCATGTTTGTGA	NM_008084
	R-GGGTGGCAGTGATGGCATGGAC	
PMCA 1 (*Atp2b1*)	F-TCAACGACTGGAGCAAGGAG	NM_001359506
	R-AAGGTCACCGTACTTCACTTGG	
PMCA 2 (*Atp2b2*)	F-TACACAGGACTCCCCTCTCAA	NM_009723
	R-CGGTTTCCTCAGAAGCAGAGT	
PMCA 3 (*Atp2b3*)	F-TGCCTGCATTACTCAGGACTC	NM_177236
	R-GAGATGAGAGGCTTGTCCCG	
PMCA 4 (*Atp2b4*)	F-GCAAAGACCCAGGATGGAGT	NM_001167949
	R-GACATGATCAGACCCGCCTT	

A list of all primer sequences used and GenBank reference numbers for the genes of interest.

### Experimental design and statistical analysis

Data are in all cases presented as mean ± SD, where replicates are individual endplates. All statistical analyses were performed in Prism 9.5 software (GraphPad). All analyses were conducted using nonparametric tests because of violations in normality of homogeneity of variance within the datasets. Paired comparisons were analyzed using Wilcoxon tests. Multiple groups were analyzed using Kruskal–Wallis (when SDs were available) or Brown–Forsythe tests (when SEs were found), followed by *post hoc* pairwise comparisons (Dunn's or Dunnett's test). For comparing two groups, Mann–Whitney tests were used.

## Results

### Validation and calibration of pHusion-Ex

**Figure 1.**
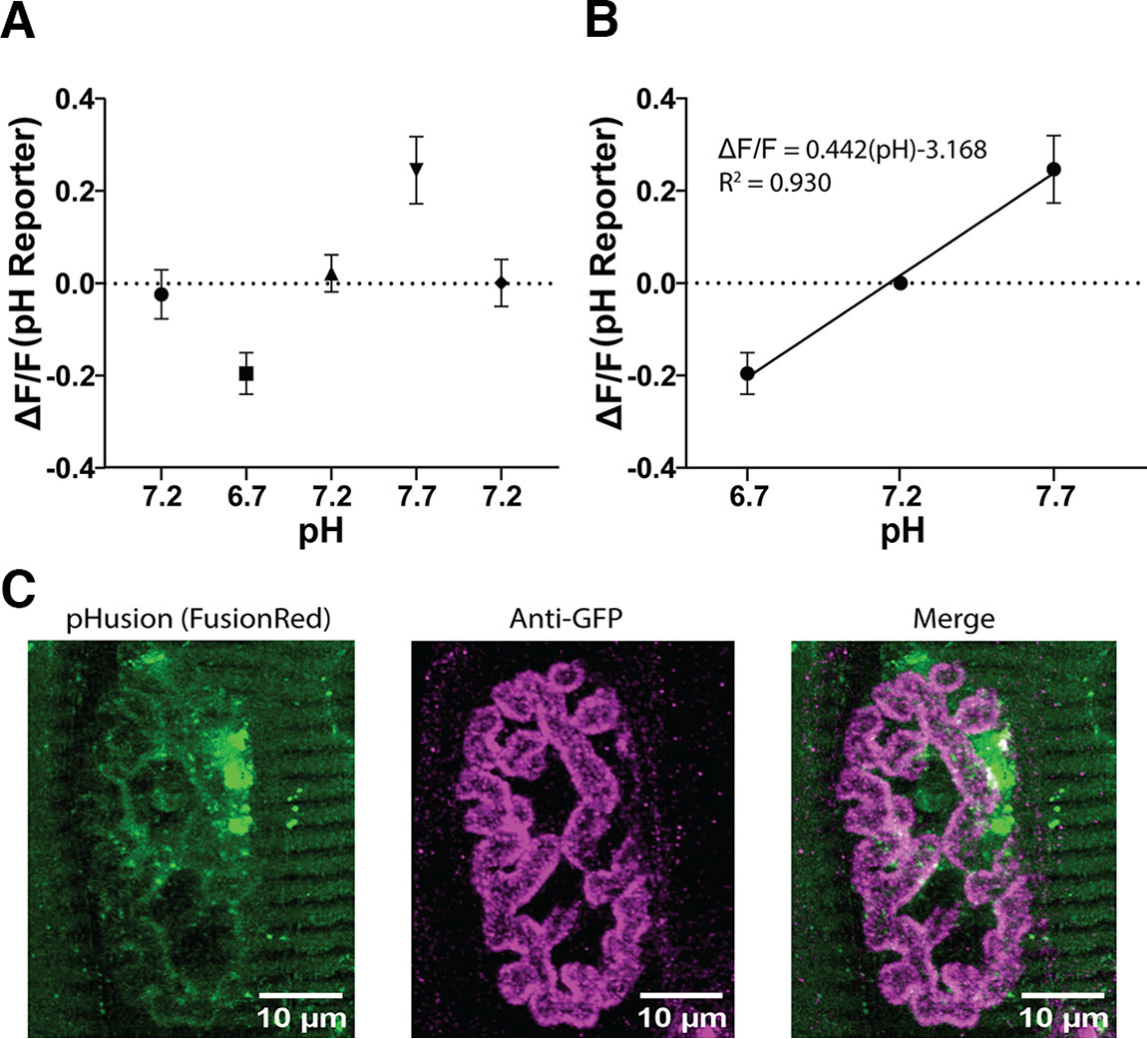
Virally expressed pHusion-Ex responds to changes in extracellular pH at the NMJ. Data are presented as mean ± SD in all graphs. ***A***, Fluorescence ratio of pHusion-Ex at various bath pHs. Levator auris longus muscles infected with AAV-pHusion-Ex were incubated in a static bath buffered with 20 mm HEPES and 10 mm HCO_3_, at indicated pH (*N* = 16). ***B***, Calibration of relationship between pH and fluorescence change, normalized to pH 7.2. ***C***, Prominent surface expression of GFP/pHusion-Ex at the NMJ endplate. Maximum *z*-projection of a confocal image stack of a levator auris longus muscle infected with pHusion-Ex and stained under nonpermeabilizing conditions with an anti-GFP antibody. Images were taken at 40×. Left (green), pHusion-ex expression; middle (magenta) anti-GFP binding; right, merged pHusion-ex and anti-GFP signal.

To calibrate changes in green to red fluorescence ratios into changes in extracellular pH, we imaged pHusion-Ex at infected NMJ endplates in buffer containing 20 mm HEPES and 10 mm HCO_3_, adjusted to pH values of 6.7, 7.2, and 7.7 (Fig. 1*A*). We saw ∼20% change in fluorescence ratio between 0.5 pH changes within the buffer, and generated a linear calibration (ΔF/F_0_ = 0.4423(pH) + 3.168; *R*^2^ = 0.9301; Fig. 1*B*). This function was subsequently used to convert green to red ratios normalized to prestimulus time points to changes in pH. We also confirmed that pHusion-Ex was expressed on the extracellular face of the sarcolemma and sampling the synaptic cleft. Using fixed but unpermeabilized tissue, anti-GFP localized to the synaptic cleft at NMJ endplates infected with pHusion-Ex, demonstrating that the probe was translocating to the membrane, expressed extracellularly, and aggregating at the synaptic cleft (Fig. 1*C*). As seen in in Figure 1*C*, there was expression of pHusion-Ex across the tissue that was not colocalized with the anti-GFP antibody. We attribute this to pHusion-Ex expression in intracellular subcompartments. To minimize the contribution of this background signal, ROIs were limited to a square area fitted around the NMJ for the analysis for all experiments.

### Alkalization of the NMJ cleft is dependent on stimulation duration and action potential–dependent signaling pathway in muscle

**Figure 2.**
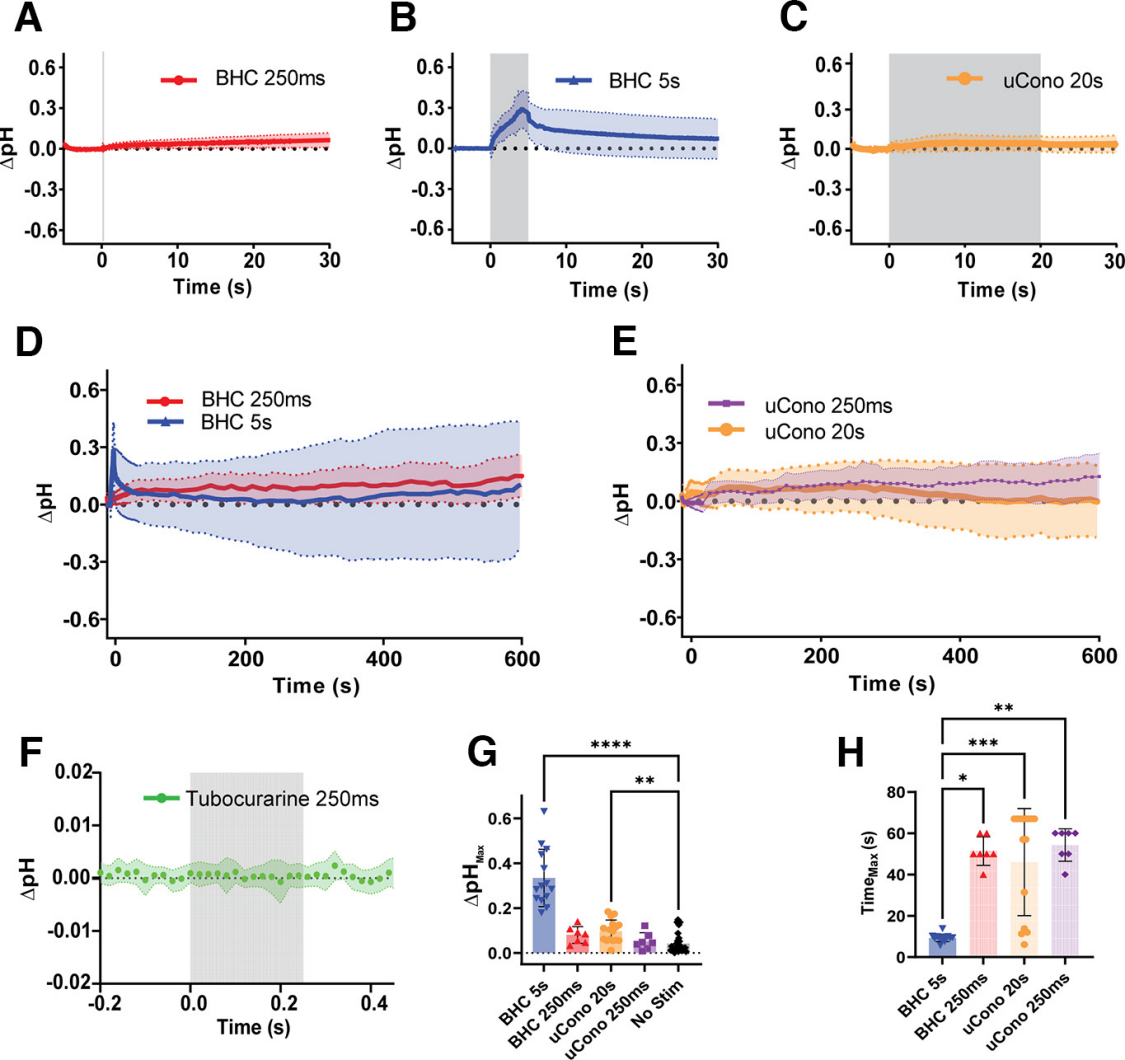
The NMJ synaptic cleft alkalizes in response to synaptic stimulation when postsynaptic signal transduction is intact. Synaptic cleft pH was monitored using muscle-expressed pHusion-Ex. Myosin inhibitor BHC blocked muscle contraction, and Na_v_1.4 antagonist μ-Conotoxin blocked muscle action potential generation. Data are presented as mean ± SD in all graphs. ***A***, An expanded timescale of the 250 ms stimulation condition in BHC, illustrating mild cleft alkalization that does not recover. The gray area (vertical line) indicates when stimulation occurred. ***B***, An expanded timescale of the 5 s stimulation condition in BHC, illustrating cleft alkalization and recovery to baseline. The gray area indicates when stimulation occurred. ***C***, An expanded timescale of the 20 s stimulation condition in µCono, illustrating slow and mild alkalization of the cleft. The gray area indicates when stimulation occurred. ***D***, Full timescale showing summary of changes in pH when incubated with BHC and stimulated for 250 ms at 50 Hz (red, *N* = 7), or 5 s at 50 Hz (blue, *N* = 14). ***E***, Full timescale showing activity-dependent changes in pH were largely absent when incubated with µCono (purple, 250 ms stimulation at 50 Hz, *N* = 7; orange, 20 s stimulation at 50 Hz, *N* = 16). ***F***, Truncated timescale of the 250 ms stimulation at 50 Hz when nAChRs are blocked with tubocurarine, displaying no change from baseline (*N* = 8). The gray area indicates when stimulation occurred. ***G***, Comparison of maximum cleft pH alkalization. Stimulation for 5 s in BHC and stimulation for 20 s in µCono were sufficient to cause significant alkalization compared with no stimulus controls (****p < 0.0001 and **p = 0.008, respectively). All other conditions did not significantly deviate from baseline, *p* > 0.25. ***H***, Comparison of the time at which maximum alkalization occurs. Simulation for 5 s, when incubated with BHC, alkalized significantly faster than all other conditions (250 ms BHC, *p = 0.014; 20 s µCono, ***p = 0.0002; 250 ms µCono, **p = 0.005) and reached a maximum during the stimulus train. All other conditions were not significantly different from one another. Stimulation onset was at 0 s.

To aid in live imaging during stimulation and limit contraction, dissected LALs were incubated with either BHC to inhibit muscle-specific myosin or µCono, a voltage-gated sodium channel (Na_v_1.4) inhibitor (Cruz et al., 1985; Li and Tomaselli, 2004). These compounds also altered Ca^2+^ dynamics, either not interfering with or inhibiting intracellular Ca^2+^ release in muscle, respectively (see above, Materials and Methods). Dissected nerves were stimulated at 50 Hz for various durations, and LAL preparations expressing pHusion-ex were imaged over the course of 600 s at ∼32°C. A 250 ms stimulation train in both BHC (*N* = 7) and µCono (*N* = 7) did not show an activity-dependent change in pH but did have a slight and prolonged increase in pH that did not recover (Fig. 2*A*,*D*,*E*). As Blaustein et al. (2020) saw acidification at the synapse, we took a closer look at the 250 ms stimulation condition. Increasing our temporal resolution, we imaged every 20 ms (50 Hz) to match the previous report. We also blocked nAChRs using 15 um tubocurarine; however, we did not extend the action potential using 3,4-DAP as in Blaustein et al. (2020). Despite increasing our temporal resolution, we did not see any cleft acidification (*N* = 8; Fig. 2*F*).

During a 5 s stimulation, endplates incubated in BHC (*N* = 14) showed a large alkalizing response to stimulation that returned to baseline shortly after stimulation ended (Fig. 2*B*). Interestingly, when we blocked movement with µCono, which blocks Na_v_1.4 and thus generation of an action potential in the muscle, and stimulated the nerve for 20 s (*N* = 16), we also observed a statistically significant alkalization, but this alkalization was markedly less than that observed when depolarization of the muscle and action potential–mediated depolarization mechanisms remained intact (Fig. 2*C*,*E*). Because of the small changes in the 20 s µCono condition and the lack of significant changes in the 250 ms µCono condition, it is unlikely that a significant pH change would occur in the 5 s µCono condition; therefore, we did not find it prudent to collect these data.

For quantitative comparison, we measured the maximum change in pH that occurred within 1 min after stimulus onset. We observed significant alkalization from baseline in muscles when incubated with BHC and stimulated for 5s (0.33 ± 0.13 pH increase, *p* < 0.0001, Dunn's test; Fig. 2*B*,*G*). In contrast, incubation with µCono showed a very small pH change when stimulated for 20 s (0.10 ± 0.05 pH increase, *p* < 0.008, Dunn's test; Fig. 2*C*,*G*). Short 250 ms stimulation trains did not cause appreciable alkalization in either BHC (ΔpH = 0.08 ± 0.04; *p* = 0.246, Dunn's test; Fig. 2*A*,*D*,*G*) or µCono (ΔpH 0.05 ± 0.04; *p* > 0.9999, Dunn's test; Fig. 2*E*,*G*). The average time to reach maximum pH increase was 4.33 ± 1.77 s during 5 s stimulation in BHC but was substantially longer for all other stimulation paradigms tested (*H* = 36.14, *p* < 0.0001, Kruskal–Wallis; Fig. 2*H*). These data suggest that alkalization of the NMJ is relatively slow and is influenced by steps in the excitation-contraction coupling cascade downstream of Na_v_1.4 depolarization of the sarcolemma. Further, these results indicate that alkalization is largely because of contributions from the postsynaptic compartment at the mammalian NMJ. We saw no change in pH when inhibiting nAChRs, and acidification was reported previously during high-frequency stimulation at room temperature when the postsynaptic contribution to cleft pH was similarly blocked (Blaustein et al., 2020).

### Strenuous stimulation leads to a biphasic pH transient in the synaptic cleft

**Figure 3.**
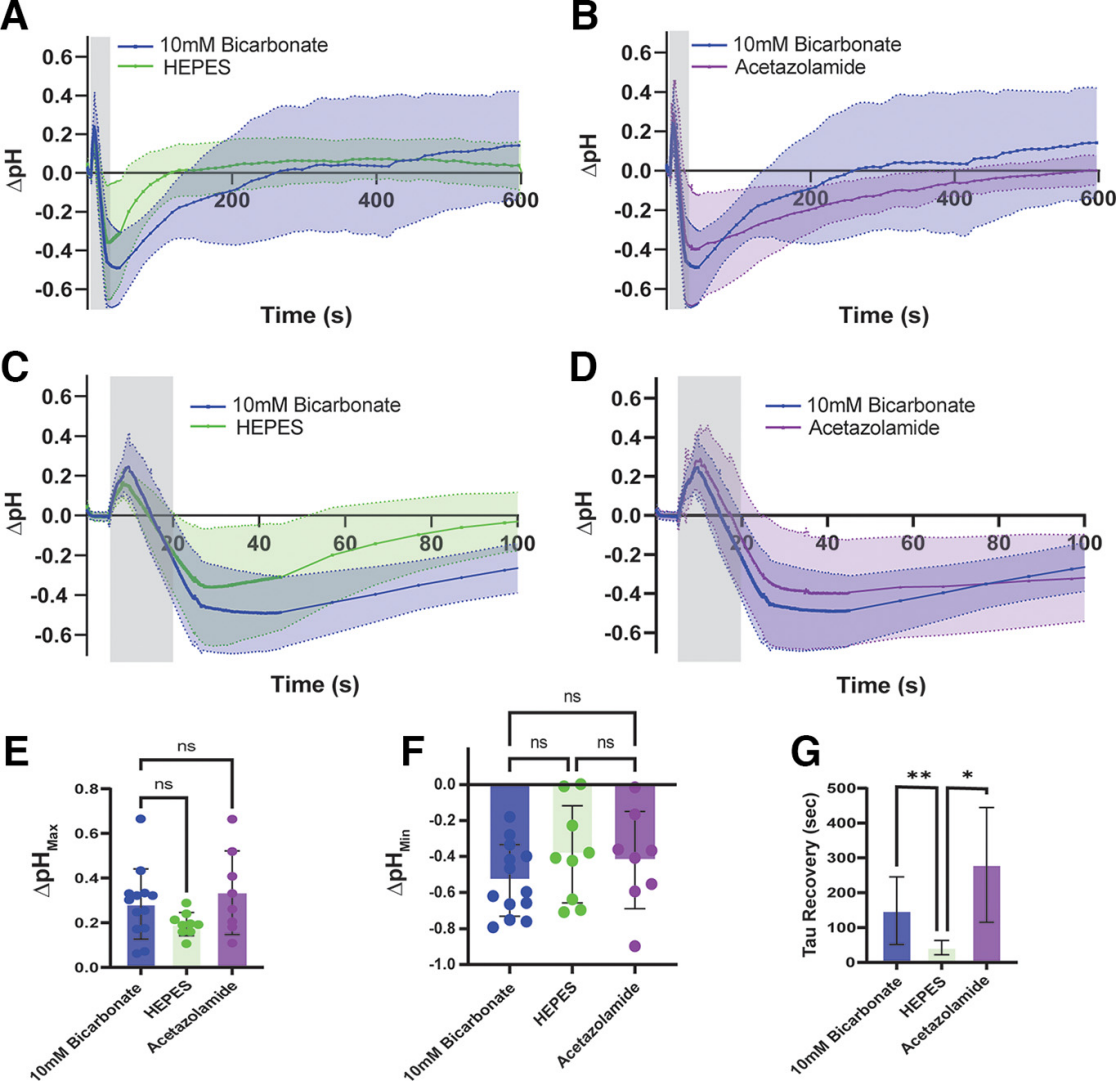
HEPES accelerates recovery from fatigue-induced acidification. Data are presented as mean ± SD in all graphs. ***A***, The time courses of preparations incubated with BHC and stimulated for 20 s at 50 Hz. The blue trace shows the summary of pH changes in endplates incubated in physiological buffer (10 mm bicarbonate, *N* = 13). The green trace shows pH changes in endplates incubated in a high-capacity pH buffer (20 mm HEPES and 10 mm bicarbonate, *N* = 9). The gray area indicates when stimulation occurred. ***B***, Time courses of preparations incubated with BHC and stimulated for 20 s at 50 Hz, assessing the role of extracellular carbonic anhydrase. The blue trace shows pH changes in endplates incubated in physiological buffer (10 mm bicarbonate) as in ***A***. The purple trace shows cells incubated with 100 μm acetazolamide to block carbonic anhydrase and limit endogenous buffering (*N* = 8). The gray area indicates when stimulation occurred. ***C***, An expanded timescale of pH changes in ***A***, showing the difference between the original (10 mm, blue) and high-capacity (20 mm HEPES, green) buffering conditions. Note the slight reduction in alkalization in the presence of high HEPES. The gray area indicates when stimulation occurred. ***D***, An expanded timescale of pH changes in 10 mm bicarbonate and 100 μm acetazolamide, showing the maximum pH changes when endplates were incubated with BHC and stimulated for 20 s at 50 Hz. The gray area indicates when stimulation occurred. ***E***, Summary comparison of maximum alkalization. HEPES buffer and treatment with 100 μm acetazolamide did not significantly reduce alkalization compared with the physiological buffer (*p* > 0.15). ns = no significant difference. Note the wide variability in the data. ***F***, Summary comparison of the maximum acidification. No significant change was measured (*p* > 0.68, Kruskal–Wallis test). ***G***, Summary comparison of recovery from acidification, measured as the time constant of the recovery curve. Cells recovered significantly faster from acidification when incubated in 20 mm HEPES buffer (**p = 0.006 vs 10 mm HCO_3_, *p = 0.014 vs acetazolamide). Acetazolamide did not vary significantly from the physiological buffer (*p* > 0.178, Brown–Forsythe test). Simulation onset was at 0 s.

Because we found alkalization occurs under moderate stimulation condition but requires postsynaptic signal transduction, we wanted to compare our results to those of previous work that monitored presynaptic intracellular pH (Zhang et al., 2010). Using the same parameters as those used previously, AAV9-pHusion-Ex–infected LALs were stimulated at 50 Hz for 20 s. When incubated in BHC, these synapses displayed a similar time to peak alkalization and similar maximum pH changes as 5 s stimulation; however, stimulation for 20 s additionally resulted in a long acidification transient starting ∼15 s after stimulation onset and only recovering after ∼5 min, suggesting a possible secondary mechanism related to muscle fatigue (Fig. 3).

Muscle activation rapidly increases glycolysis, thus lactic acid by-product and extrusion from muscle could be responsible for cleft acidification. We tested for increased lactate export by blocking monocarboxylate transporters, using both α-cyano-4-hydroxycinnamic acid at 200 μm and 4 mm and AR-C155858 at 1 μm, but there was no effect versus controls (data not shown), suggesting lactate extrusion following glycolysis was not responsible for acidification (Lucas et al., 2018). Na/H exchanger (NHE) inhibitors (ethylisopropyl amiloride) and glycolysis blockers (iodoacetic acid) were also tested at various concentrations, but the integrity of the preparation was compromised by these compounds (data not shown), thwarting further exploration of these mechanisms.

Given the ATP demands of this extended stimulation protocol, it was surprising that there was no effect on acidification by inhibiting transport of lactate through monocarboxylate transporters. Despite glycolysis being the primary energy source for these fibers, prolonged stimulation may be increasing the amount of aerobic activity within the muscle during recovery, so we also tested whether acidification could be a result of CO_2_-buffering mechanisms. Carbonic anhydrase catalyzes CO_2_ and H_2_O into carbonic acid, which further dissociates into protons and HCO_3_^−^. Given the relatively long period of stimulation, the muscle may be generating excess CO_2_ through cellular respiration and driving the creation of more carbonic acid in the cleft, lowering the pH of the cleft. To test these changes, we manipulated carbonate buffer capacity of the bath solution. First, we added a high concentration of a phosphate buffer, HEPES, to our 10 mm HCO_3_ physiological solution to increase the general buffering capacity and test whether these changes in pH are susceptible to extracellular buffering. Increasing buffer capacity by adding 20 mm HEPES did not significantly decrease alkalization but sped recovery from acidification (Fig. 3*A*,*C*). We then reduced buffer capacity by impairing the CO_2_ buffering system. Specifically, we blocked carbonic anhydrase with a 20 min incubation in 100 um acetazolamide; however, this inhibitor only had a marginal effect on pH transients (Fig. 3*B*,*D*). Comparing the maximum changes in pH over the first minute, 20 mm HEPES (ΔpH = 0.19 ± 0.05, *N* = 9) reduced alkalization but did not reach statistical significance when compared with the 20 s stimulation controls in 10 mm HCO_3_ (ΔpH = 0.28 ± 0.16, *N* = 13; *H* = 4.32, *p* = 0.155, Kruskal–Wallis test; Fig. 3*E*). Peak acidification was reduced in 20 mm HEPES (ΔpH = −0.39 ± 0.27) but was also not statistically significant from controls (ΔpH = −0.53 ± 0.20; *H* = 2.09, *p* = 0.685, Kruskal–Wallis test; Fig. 3*F*). The lack of effect may be because of variability of responses in the control condition that was not seen when incubating with HEPES (Fig. 3*E*,*F*). Acetazolamide did not have any effect on the maximum changes in alkalization (ΔpH = 0.33 ± 0.19, *N* = 8; *p* > 0.9999, Dunn's test) or acidification (ΔpH = −0.42 ± 0.27; *p* > 0.9999, Dunn's test; Fig. 3*E*,*F*). However, 20 mm HEPES significantly sped recovery from acidification, fit by a single exponential (τ = 42.56 ± 20.48 s, F* = 9.37, *p* = 0.0055, Brown–Forsythe test; Fig. 3*G*) when compared with all other conditions (10 mm HCO_3_, τ = 148.5 ± 96.92 s; acetazolamide, τ = 280.1 ± 164.9 s). Acetazolamide slowed recovery time slightly but was not statistically significant from other conditions (*p* > 0.178, Dunnett's test; Fig. 3*G*). The observation that acetazolamide did not significantly affect the transient suggests membrane-bound carbonic anhydrase in the synaptic cleft does not contribute substantially to buffering capacity at the synaptic cleft of the vertebrate NMJ; however, soluble carbonic anhydrase was not included in our buffer, limiting interpretation. Although carbonic anhydrase has been identified in the sarcolemma, it may not be located near the endplate or its expression may be muscle-fiber specific (Geers et al., 1985). Overall, these data suggest that extracellular buffering of the NMJ cleft is limited, possibly because of an inhibited buffer diffusion between the synaptic cleft and bulk extracellular media caused by the basal lamina that divides the presynaptic and postsynaptic side of the synapse (Patton, 2003). The possibility of structures in the synaptic cleft having an impact on buffering was suggested in computational models of the *Drosophila* NMJ, suggesting that synaptic structures closed off to diffusion strongly reduce cleft buffering (Feghhi et al., 2021). More data are necessary to understand this phenomenon.

### Intracellular Ca^2+^ load in muscle is inhibited by µ-Conotoxin

**Figure 4.**
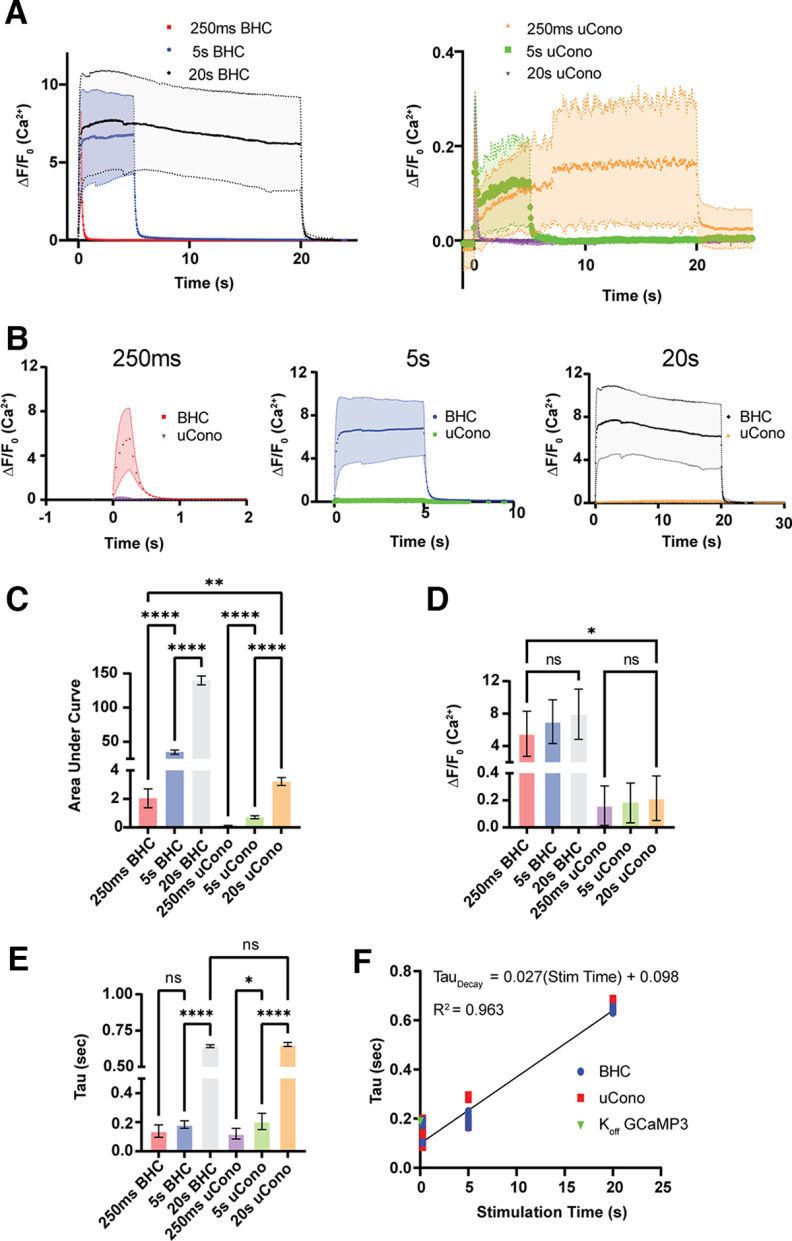
Muscle-expressed GCAMP shows a significantly larger cytosolic Ca^2+^ transient in BHC when compared with µCono. Data are presented as mean ± SD in all graphs. ***A***, Summary of changes in GCaMP3 signal in response to 250 ms, 5 s, and 20 s stimulation trains at 50 Hz in either BHC (left, *N* = 9) or µCono (right, *N* = 10). Note the difference in response magnitude between graphs. ***B***, Comparison of fluorescent GCaMP3 signal in response to 250 ms (left), 5 s (middle), and 20 s (right) in BHC versus µCono. ***C***, Summary comparison of the integral of GCaMP3 response. The area under the curve for all conditions was significantly different from each other (all other conditions, *p* < 0.021; Dunnett's test). ns = no significant difference, *p < 0.5, **p < 0.01, ***p < 0.001, ****p < 0.0001. ***D***, Summary comparison of maximum fluorescence values. All BHC conditions had significantly higher maximums than all µCono conditions (BHC vs µCono, *p* < 0.05; Dunn's test) Similar peak GCaMP3 signal in BHC was seen across all stimulation durations. Similarly, µCono peak responses were independent of stimulation duration. ***E***, Summary comparison of GCaMP signal decay following stimulation. Decay of GCaMP fluorescence after stimulation ended was fit with a single exponential. Time constant for recovery was significantly slowed by increasing stimulus duration (250 ms BHC vs 5 s BHC, *p* = 0.2; 250 ms BHC vs 5 s µCono, *p* = 0.12; all other time comparisons, *p* < 0.05, Dunnett's test). Stimulus trains with the same duration were not significantly different between BHC and µCono (*p* > 0.999, Brown–Forsythe test). ***F***, Regression line relating the time constant of recovery to stimulation time (0 calculated from the *k*_off_ = 5.3 s^−1^ of GCaMP3). Simulation onset was at 0 s.

We next aimed to clarify the mechanism underlying transient alkalization at NMJs in BHC during 5 s and 20 s stimulation trains, and whether this was linked to liberation of intracellular Ca^2+^. Extracellular alkalization in other cell types depends on Ca^2+^ flux, particularly Ca^2+^ extrusion via the PMCA (Makani and Chesler, 2010; Feghhi et al., 2021). To correlate Ca^2+^ dynamics with pH transients at the LAL NMJ, we used mice expressing GCaMP3 in muscle to follow Ca^2+^ transients during 50 Hz stimulation trains (Fig. 4*A*,*B*). We inferred relative levels of labile intracellular Ca^2+^ based on the integral of the fluorescence time series (Fig. 4*C*). Not surprisingly, the area under the curve was greater in BHC conditions (250 ms, 2.04 ± 0.67; 5 s, 35.00 ± 3.06; 20 s, 139.70 ± 6.38; *N* = 9) when compared with µCono conditions (250 ms, 0.11 ± 0.03; 5 s, 0.70 ± 0.10; 20 s, 3.05 ± 0.27; *N* = 10), and increased based on stimulus duration (F* = 3294, *p* < 0.0001, Brown–Forsythe test). Further, peak Ca^2+^ increase was higher in NMJs incubated in BHC (250 ms, ΔF/F_0_ = 5.52 ± 2.77; 5 s, ΔF/F_0_ = 7.02 ± 2.70; 20 s, ΔF/F_0_ = 7.93 ± 3.10) than those incubated in µCono (250 ms, ΔF/F_0_ = 0.16 ± 0.15; 5 s, ΔF/F_0_ = 0.19 ± 0.14; 20 s, ΔF/F_0_ = 0.21 ± 0.17; *H* = 43.38; *p* < 0.0001, Kruskal–Wallis test; Fig. 4*D*). Although endplates specifically were measured, it was clear that the distribution of signal was different depending on which drug the LAL was incubated with. In the case of BHC, the large Ca^2+^ signal seemed to diffuse and remain relatively uniform across the NMJ and surrounding fiber in the field of view. When incubated with µCono, the smaller Ca^2+^ signal was restricted to the endplate. The recovery from stimulus, as measured by the rate of decay to baseline after the cessation of stimulation, was also progressively slowed by extending stimulation duration equally in both BHC and µCono conditions (250 ms, τ_Decay_ = 0.11 ± 0.04; 5 s, τ_Decay_ = 0.17 ± 0.02; 20 s, τ_Decay_ = 0.60 ± 0.06), with the exception of the 250 ms BHC condition, which was not significant from the 5 s conditions (250 ms BHC vs 5 s BHC, *p* = 0.199, 250 ms vs 5 s µCono, *p* = 0.119, Dunnett's test; Fig. 4*E*). This result suggests that Ca^2+^ buffering mechanisms in muscle have a temporal component that limit buffering, independent of Ca^2+^ load (F* = 504.5, *p* < 0.0001, Brown–Forsythe; Fig. 4*E*). Using the *k*_off_ (5.3 s^−1^) of GCaMP3 at the physiological temperature reported by Helassa et al. (2015) to find the dissociation constant for 0 s of stimulation, we used a linear regression to establish the relationship between stimulation time and decay of Ca^2+^ signal (τ_Decay_ = 0.027 * Stim Time +0.098, *R*^2^ = 0.963; Fig. 4*F*). One possible explanation for the slowing of Ca^2+^ decay could be the slow binding to parvalbumin following Mg^2+^ dissociation during prolonged tetanus (Westerblad and Allen, 1994; Nogueira et al., 2022), but this line of inquiry was not pursued further. In summary, these data indicate Na_v_1.4 is needed to boost the amplitude of labile intracellular Ca^2+^ in muscle and that Ca^2+^ load and handling can be affected by stimulation duration. An accumulation of Ca^2+^ in µCono is likely because of Ca^2+^ conductance through nAChRs (Shen and Yakel, 2009). The results suggest the difference in rapid cleft alkalinization during 5 s stimulation in BHC, and slower smaller alkalinization in 250 ms BHC or 20 s in µCono (Fig. 3*G*,*H*), may indicate a minimum threshold of intracellular Ca^2+^ accumulation needed to initiate alkalizing mechanisms.

### Increasing intracellular Ca^2+^ release during stimulation increases cleft pH

**Figure 5.**
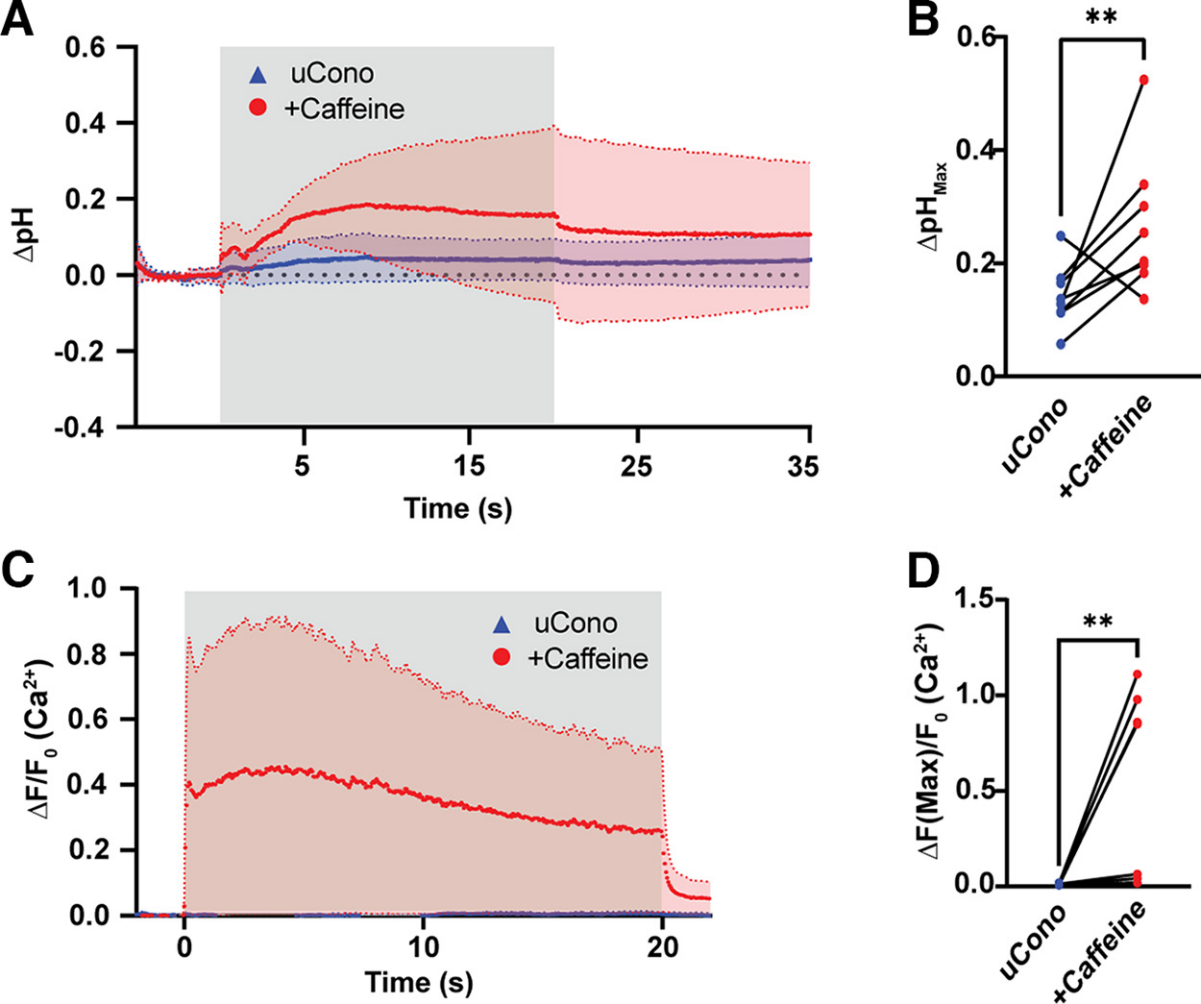
Alkalization is modulated by intracellular Ca^2+^ release from the sarcoplasmic reticulum. Data are presented as mean ± SD in all graphs. ***A***, Cleft pH changes are enhanced in µCono-treated preparations by the ryanodine receptor agonist caffeine (*N* = 8, red trace) when stimulated for 20 s at 50 Hz. Cleft pH in µCono alone had minimal alkalinization (paired and nonpaired data shown; *N* = 16, blue trace). The gray area indicates when stimulation occurred. ***B***, Summary pairwise comparison shows increased pH change because of caffeine; **p = 0.007. ***C***, GCaMP3 signal is increased after exposure to caffeine (*N* = 8, red trace). Virtually no GCaMP3 signal was seen because of stimulation in µ-Conotoxin (*N* = 8, blue trace). The gray area indicates when stimulation occurred. ***D***, Maximum normalized GCaMP3 fluorescence was significantly increased by caffeine; **p = 0.008.

In muscle, depolarization is accompanied by release of Ca^2+^ from intracellular stores, a mechanism needed to facilitate contraction (Endo, 2009). Blocking nAChRs in previous studies, or Na_v_1.4 activation as shown above, blocks muscle action potential generation and precludes intracellular Ca^2+^ liberation; however, in BHC this signal transduction mechanism remains intact. To bypass the effect of lower intracellular Ca^2+^ release and determine whether cleft alkalization was dependent on this release, we attempted to boost sensitivity of store-operated Ca^2+^ release pharmacologically. Caffeine has been used previously to increase intracellular Ca^2+^ release in muscle by stimulating ryanodine receptor activity (Sokolow et al., 2004; Allen et al., 2008). We added 5 mm caffeine to the bath in µCono-treated preparations and stimulated after an ∼20 min incubation period in paired experiments where images of pHusion-Ex NMJ endplates were first acquired without caffeine (Fig. 5). Although baseline pH was not affected, stronger alkalization was induced by adding caffeine (ΔpH = 0.267 ± 0.123, *N* = 10) over controls (ΔpH = 0.142 ± 0.056, *N* = 10), increasing the maximum pH by ∼0.12 and almost doubling the previous pH change in response to a 5 s stimulation train (*U* = 7, *p* = 0.007, Mann–Whitney *U* test; Fig. 5*A*,*B*). To verify that Ca^2+^ was also increased by caffeine in these experiments, we performed a similar stimulation protocol and visualized GCaMP3-expressing NMJs. Intracellular Ca^2+^ concentrations in µCono were increased nearly 40-fold by caffeine incubation (Caffeine, ΔF/F_0_ = 0.493 ± 0.495, *N* = 8; Control, ΔF/F_0_ = 0.013 ± 0.004, *N* = 8; *W* = 36, *p* = 0.008, Wilcoxon test; Fig. 5*C*,*D*). These data suggest that liberation of Ca^2+^ from intracellular stores of muscle is responsible for cleft pH alkalization.

### PMCA is localized to the cleft and contributes to stimulation-dependent alkalization

**Figure 6.**
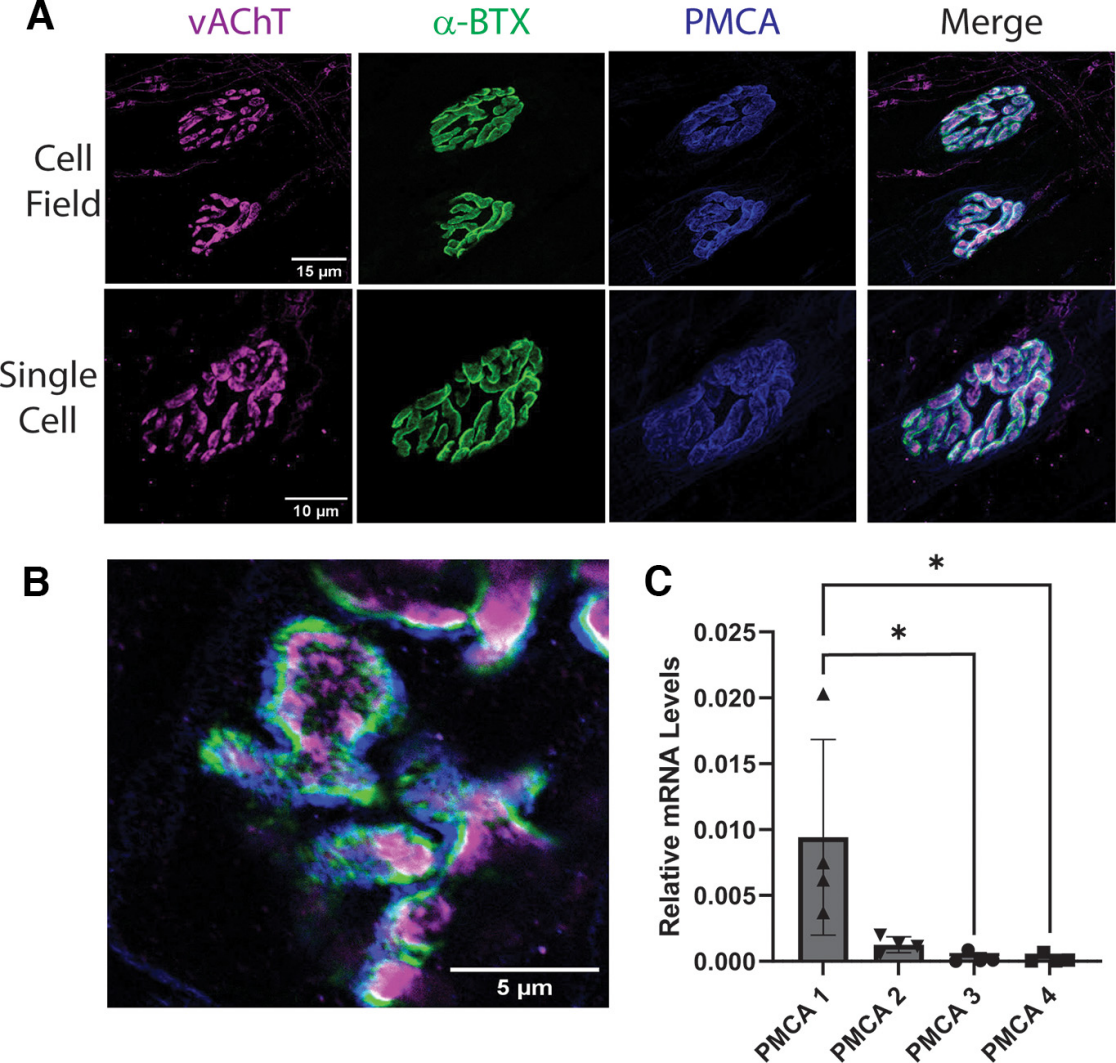
PMCA localizes to the NMJ endplate. ***A***, Example maximum *z*-projections of deconvolved confocal images of NMJ endplates show close correlation between PMCA and synaptic markers. Magenta, anti-vAChT; green, Alexa Fluor 594 conjugated to α-bungarotoxin; blue, anti-pan-PMCA. ***B***, Three-dimensional-STED image of a portion of an LAL endplate shows PMCA is located extrasynaptically, adjacent to nAChRs. Lateral resolution is 60–100 nm in these images. ***C***, Transcript abundance of PMCA isoforms (*Atp2b1-4*) relative to GAPDH was determined by qRT-PCR from LAL lysate. PMCA1 is transcribed significantly more than PMCA3 and PMCA4; *p = 0.037 for both comparisons, but not significantly more than PMCA2, *p* = 0.602 (Dunn's test). All other comparisons were not significantly different (*p* > 0.602); *N* = 4 independent replicates (animals). Data are presented as mean ± SD.

Because labile intracellular Ca^2+^ seems to be linked to alkalization of cleft pH, we attempted to causally relate it via PMCA activity, a mechanism proposed for alkalization of central synapses (Makani and Chesler, 2010). Little is known about PMCA expression in muscle, and specifically whether it is present at the NMJ endplate (Hammes et al., 1994; Sacchetto et al., 1996). We labeled the PMCA in the LAL via immunohistochemistry with a pan-PMCA antibody in fixed tissue and determined it is expressed at the NMJ endplate (Fig. 6). In this experiment, fluorescently labeled α-bungarotoxin was used to stain nAChRs as a postsynaptic marker, and antibodies against vAChTs were used as a presynaptic marker (Fig. 6*A*). Surprisingly, we found that PMCA is expressed almost exclusively at the NMJ endplate in the LAL, adjacent to presynaptic and postsynaptic compartments, and shows very limited spatial overlap with either vAChTs or nAChRs (Fig. 6*B*). Of the four known PMCA isoforms in mouse (*Atp2b1-4*), we determined via qRT-PCR that PMCA1 is the dominant transcript in LAL tissue (mean abundance relative to GAPDH = 0.94 ± 0.74%), with a very minor contribution from PMCA2 (mean abundance relative to GAPDH = 0.13 ± 0.06%; *N* = 4 mice, χ^2^ = 10.80, *p* = 0.002, Friedman test; Fig. 6*C*). PMCA isoforms 3 and 4 were not detected.

**Figure 7.**
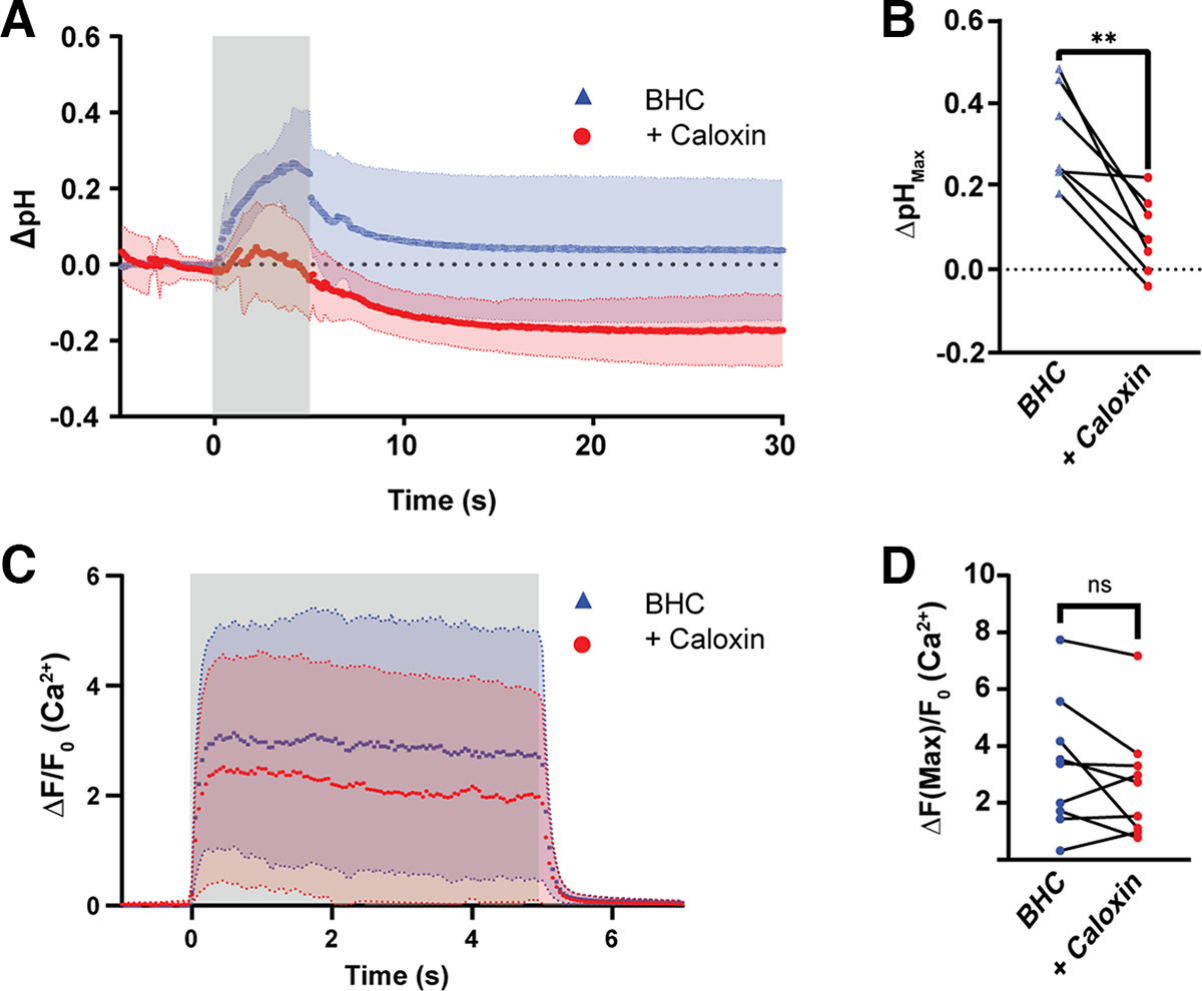
Alkalization at the neuromuscular junction requires PMCA activity. Data are presented as mean ± SD in all graphs. ***A***, In paired experiments, synaptic cleft pH changes were muted by the PMCA inhibitor caloxin 1b1 (*N* = 7, red) compared with paired and nonpaired control cells in BHC (*N* = 14, blue) when stimulated for 5 s at 50 Hz. The gray area indicates when stimulation occurred. ***B***, Summary of maximum alkalization (*N* = 7 cells) in paired experiments before and during caloxin exposure. Cleft alkalization was significantly reduced by caloxin, **p = 0.0012. ***C***, Caloxin marginally attenuated GCAMP3 signal (*N* = 10, blue, BHC; *N* = 10, red, BHC + caloxin). The gray area indicates when stimulation occurred. ***D***, Maximum normalized GCaMP fluorescence was not affected by caloxin in paired experiments.

As PMCA was localized at the endplate synaptic cleft, and alkalization is dependent on accumulation of intracellular Ca^2+^, we then determined whether PMCA is responsible for alkalization of the synaptic cleft. We inhibited PMCA with focal perfusion of the peptide inhibitor caloxin 1b1 onto endplates in LAL preparations. In paired experiments, the LAL was stimulated with a 5 s train at 50 Hz in BHC, followed by local perfusion of 500 um caloxin 1b1 in physiological solution onto endplates being ejected continuously from a glass pipet with a 15–30 μm pipet tip for ∼5 min before and during a second imaging bout. Blocking the PMCA strongly reduced the resulting pH transient (Fig. 7*A*). In caloxin 1b1, cleft alkalization was strongly suppressed (ΔpH = 0.08 ± 0.09, *N* = 7) compared with paired within-cell controls (ΔpH = 0.32 ± 0.12, *N* = 7; *U* = 1, *p* = 0.001, Mann–Whitney *U* test; Fig. 7*B*). Confirming cleft alkalization was because of PMCA activity and that caloxin did not block intracellular Ca^2+^ liberation, the experimental protocol was repeated in GCaMP3-expressing NMJs, which showed no significant difference in intracellular Ca^2+^ concentration because of caloxin treatment (*N* = 10, ΔF/F_0_ = 2.51 ± 1.98) compared with within-cell controls (*N* = 10, ΔF/F_0_ = 3.16 ± 2.23; *W* = 27, *p* = 0.19, Wilcoxon test: Fig. 7*C*,*D*). These results suggest that the inhibition of PMCA does not affect overall Ca^2+^ buffering or other Ca^2+^ clearance pathways but that PMCA activity mediates activity-dependent alkalization of the mammalian NMJ synaptic cleft.

## Discussion

In this study we investigated pH transients during activity at the mouse NMJ using a commonly used fast-twitch muscle fiber synapse preparation (Erzen et al., 2000; Tabares et al., 2007; Zhang et al., 2010; Ruiz et al., 2011; Wright et al., 2011; Burke et al., 2018; Blaustein et al., 2020; Ojeda et al., 2020). Taking advantage of a muscle-specific myosin blocker to inhibit contraction, we were able to examine the effects of activity in near-physiological conditions, with the postsynaptic transduction cascade intact in muscle. Consistent with other excitatory synapses, we found activity-induced alkalization of the synaptic cleft (Chen and Chesler, 2015; Stawarski et al., 2020). Surprisingly, we also observed a sustained large acidification of the synaptic cleft that lasted minutes when the synapse was driven toward fatigue.

### Mechanism of cleft alkalization

We showed synaptic alkalization is dependent on postsynaptic liberation of Ca^2+^. Muscle action potentials result in sarcoplasmic release of Ca^2+^ (Endo, 2009; Calderón et al., 2014). We pharmacologically blocked cleft alkalization with inhibitors of muscle action potential generation (Na_v_1.4; Fig. 2), and could rescue synaptic cleft alkalization by augmenting the Ca^2+^ transient with caffeine (Fig. 5), indicating the source of postsynaptic Ca^2+^ is likely release from intracellular stores. Alkalization of the cleft was dependent on PMCA extrusion of muscle Ca^2+^ as the allosteric PMCA inhibitor caloxin 1b1 muted the pH rise (Fig. 7*A*,*B*). Notably, there was no change in labile Ca^2+^ load when PMCA was inhibited (Fig. 7*C*,*D*), indicating other mechanisms in muscle are responsible for Ca^2+^ buffering from the cytosol. Given that sarcoendoplasmic reticulum Ca^2+^-ATPase is the primary route for Ca^2+^ buffering in excitable cells (Calderón et al., 2014; Stafford et al., 2017), we presume PMCA plays a marginal role in bulk cytosolic Ca^2+^ extrusion.

The highly organized arrangement and spatial localization of PMCA at the NMJ endplate is consistent with PMCA aggregating near synapses, as established at glutamatergic synapses (DeMarco and Strehler, 2001; Burette et al., 2010). Functionally, this tight association with the endplate may focus Ca^2+^ extrusion into the synaptic cleft and causes cleft alkalization. This result is mirrored by findings in *Drosophila* NMJ, where cleft pH transients measured from the postsynaptic side of the NMJ cleft were larger than those from the presynaptic side, implicating a large contribution of postsynaptic Ca^2+^ buffering to cleft pH, presumably via PMCA (Stawarski et al., 2020). Why and how these pumps are concentrated on the postsynaptic membrane surrounding the endplate is unknown. Extracellular alkalization has been shown to delay muscle fatigue during exercise (Sostaric et al., 2006), so localized PMCA activity may confer some benefit to fast-twitch muscle function. Muscle fiber type may influence different responses to pH change, and slow-twitch muscle may show different PMCA localization and cleft pH transients. Further investigation in other muscle types may provide greater insight into the modulation of transmission at the NMJ and impact of disease.

### Blocking PMCA uncovers cleft acidification: potential mechanisms

Prolonged synaptic cleft acidification was observed following strenuous stimulation and also appeared during caloxin treatment (Fig. 7*A*) but not when PMCA function was intact (Fig. 5*A*), implying a constitutive proton flux during or following stimulation that is largely obscured by PMCA activity. Blocking PMCA resulted in activity-dependent acidification; however, we have yet to isolate the cause. This suggests that there may be an uncharacterized off-target effect of the PMCA inhibitor used or that acidification is intrinsically tied to cleft alkalization and fatigue.

PMCA interacts with various kinases like membrane-associated guanylate kinase and Ca^2+^/calmodulin-dependent serine protein kinase (Stafford et al., 2017) that may be causing stimulation-dependent acidification of the cleft. For example, it can bind to NHE factor 2, a protein instrumental in Ca^2+^ dependent inhibition of NHE through protein kinase A, which phosphorylates PMCA at a site made accessible by the binding of calmodulin (DeMarco et al., 2002; Baggaley et al., 2007; Stafford et al., 2017). If caloxin inhibits PMCA binding to these proteins because of restricted conformational changes, it could increase NHE activity and export of protons into the cleft through binding to NHE factor 2 or increased cytosolic Ca^2+^. Activation of the Na^+^/H^+^ exchanger has caused long-term intracellular alkalization in cultured Madin–Darby canine kidney cells when another Ca^2+^ extrusion mechanism, the Na^+^/Ca^2+^ exchanger, was inhibited (Dreval et al., 2005). Better understanding of this link may bring insight to motor diseases that show increased acidification (Friese et al., 2007; Dodge et al., 2013).

The biphasic pH transient in the cleft during prolonged stimulation mirrored intracellular changes seen in motor neurons innervating the LAL (Zhang et al., 2010). The previous study describes a short acidification that peaked 3–4 s after stimulation and a long alkalization transient in the cytosol of motor neurons at the LAL. Notably, these experiments were performed in curare and examined only presynaptic contribution. Zhang et al. (2010) postulated that presynaptic PMCA activity acidified the cytosol, followed by alkalization of the terminal because of membrane-associated vATPase from stranded synaptic vesicles pumping protons out of the neuron, acidifying the synaptic cleft. We did not observe these predicted changes in our experiments when postsynaptic action potential generation was blocked with µCono, making it unlikely vATPase or accumulated protons released by synaptic vesicle fusion contributed to transients we measured.

Two possible scenarios may reconcile these observations. First, the NMJ synaptic cleft is wide enough or sufficiently isolated by the basal lamina to contain different proton concentrations on either side of the synapse. Rapid proton diffusion out of the cleft would quickly dissipate these local gradients, like those caused by vesicular release, originating at the membrane. Facilitated diffusion or reuptake by muscle, neurons, or perisynaptic Schwann cells could cause disparities in pH buffering, but these disparities have not been described. Notably, we did not see PMCA expression in perisynaptic glia (Fig. 6; data not shown). Measurements from the *Drosophila* NMJ support local pH gradients as the magnitude of pH transients measured with postsynaptically expressed pHusion-Ex were larger than those reported from presynaptic expression (Stawarski et al., 2020). A second possibility is that presynaptic PMCAs, and potentially vATPases, are localized outside the synaptic cleft on presynaptic membrane and therefore not contributing to the cleft proton gradient. Regardless, the direction and magnitude of both postsynaptic and presynaptic pH transients are similar, although our results suggest they are driven by different processes.

### Comparison to previous work

Our results suggest a negligible contribution to cleft transients from the motor neuron terminals under physiological stimulation conditions, as shown by a lack of pH transients in µCono. We did observe a slow alkalization following a 20 s stimulus train, which may be because of the Ca^2+^ influx from Ca^2+^-permeable nAChRs and subsequent extrusion from the PMCA (Shen and Yakel, 2009). Our data stand in contrast with previous measurements at this synapse that showed acidification of the synaptic cleft at room temperature and very high frequency (300 Hz) and under very low pH buffering conditions (0.8 mm HEPES; Blaustein et al., 2020). Computational models support a short (50–500 μs) but significant initial acidification because of vesicular release that is sensitive to buffering (Feghhi et al., 2021). Although not observed, a fast transient acidification could precede synaptic cleft alkalization at the LAL. Notably, we did not observe any pH changes during a short 250 ms stimulus train (Fig. 2).

Alkalization was found at the *Drosophila* NMJ when blocking fibers with ryanodine in *ex vivo* preparation, and a nearly 10-fold greater alkalization was found *in vivo* with restrained larvae (Stawarski et al., 2020). Although it was suggested that these changes were because of different buffering capacities between experiments, our results suggest the larger pH transient could be because of intracellular Ca^2+^ release that occurs *in vivo*. Although ryanodine inhibits intracellular Ca^2+^ release, it allows action potential generation that increases intracellular Na^+^. Influx of Ca^2+^ through VGCCs during the action potential, and reversal of the Na^+^/Ca^2+^ exchanger because of increased intracellular Na^+^ (Rasgado-Flores et al., 1989), may result in an intermediate phenotype, which would not be observed in µCono. Ultimately, it seems that the degree to which excitation–contraction coupling remains intact strongly influences the extracellular pH transient.

### Experimental limitations

Although BHC largely inhibits myosin activity and muscle contraction, small movements during stimulation had an impact on our ability to resolve local transients within the endplate. Even marginal drift in the *z*-axis incapacitated movement correction algorithms. In many of these cases, image registration was performed manually. Movement artifacts limited our ability to measure subregions of endplates, so all analyses relate to average changes across entire NMJs. Second, nonlinear photobleaching of pHusion-Ex fluorophores occurred during our experimental protocols. Even though we took care to limit photoxicity and corrected for bleaching with interleaved no-stimulation controls, a reduced signal may have resulted in traces where data did not return to baseline. Additionally, rigorous stimulation induced by the 20 s protocols caused the NMJ to fail permanently in many cases. For a majority of those experiments, only one stimulation sweep was used.

### Conclusion

We identified a novel biphasic pH transient at the NMJ—both a short alkalization transient in response to moderate stimuli and a biphasic pH transient that ended with a persistent acidification transient following more prolonged stimulation of the muscle fiber. Extracellular acidification is well characterized as a sign of fatigue in muscle (Street et al., 2001; Juel, 2008) and may be indicative of a pathologic state that causes reduced neurotransmission and subsequent activation of compensatory mechanisms (Urbano et al., 2014; Zhu et al., 2021). In contrast, activity-dependent alkalization at the NMJ and its possible downstream effects on function have not been previously described. Future experiments targeting the nature of fatigue-related acidification at the cleft and impact on synaptic function may provide insight into its role at the NMJ.
